# Neuroinflammatory Dysfunction of the Blood–Brain Barrier and Basement Membrane Dysplasia Play a Role in the Development of Drug-Resistant Epilepsy

**DOI:** 10.3390/ijms241612689

**Published:** 2023-08-11

**Authors:** Yulia Zabrodskaya, Natalia Paramonova, Anastasia Litovchenko, Elena Bazhanova, Aleksandr Gerasimov, Darya Sitovskaya, Victoria Nezdorovina, Svetlana Kravtsova, Stanislav Malyshev, Ekaterina Skiteva, Konstantin Samochernykh

**Affiliations:** 1 Polenov Neurosurgical Institute—Branch of the Almazov National Medical Research Centre, 197341 St. Petersburg, Russia; apgerasimow@rambler.ru (A.G.); daliya_16@mail.ru (D.S.); dr_victoria@list.ru (V.N.); kravtsovasv@mail.ru (S.K.); malyshev.stm@gmail.com (S.M.); ski.ek.nik@gmail.com (E.S.); neurobaby12@gmail.com (K.S.); 2 Sechenov Institute of Evolutionary Physiology and Biochemistry, Russian Academy of Sciences, 194223 St. Petersburg, Russia; natapa@bk.ru (N.P.); anastasiya_litovchenkos@list.ru (A.L.); bazhanovae@mail.ru (E.B.); 3 State Research Testing Institute of Military Medicine of the Ministry of Defense of the Russian Federation, 195043 St. Petersburg, Russia; 4 Golikov Research Center of Toxicology, 192019 St. Petersburg, Russia; 5 State Scientific Center of the Russian Federation, Institute of Biomedical Problems of the Russian Academy of Sciences, 123007 Moscow, Russia

**Keywords:** drug-resistant epilepsy, blood–brain barrier, neuroinflammation, cytokines, VEGF, basement membrane, dysplasia, immunohistochemistry, electron microscopy, multiplex analysis

## Abstract

Drug-resistance epilepsy (DRE) is a key problem in neurology. It is possible that damage to the blood–brain barrier (BBB) may affect resistance in DRE. The aim of this work was to assess the damage and dysfunction in the BBB in the area of epileptic foci in patients with DRE under conditions of neuroinflammation. The changes to the BBB in temporal lobe epilepsy (by immunohistochemistry and transmission electron microscopy), levels of neuroinflammatory proteins, and cytokine levels in the blood (by multiplex analysis) were studied. Increased levels of vascular endothelial growth factor (VEGF) and growth-regulated protein (GRO), and decreased levels of epidermal growth factor (EGF) in plasma, combined with overexpression of the VEGF-A receptor by endotheliocytes were detected. Malformation-like growths of the basement membrane of the capillaries of the brain complicate the delivery of antiepileptic drugs (AEDs). Dysplasia of the basement membrane is the result of inadequate reparative processes in chronic inflammation. In conclusion, it should be noted that damage to the microcirculatory network of the brain should be considered one of the leading factors contributing to DRE.

## 1. Introduction

Epilepsy is a chronic neurological disease that affects more than 70 million people worldwide [1]. Despite the presence of more than 20 anticonvulsants for the symptomatic treatment of epileptic seizures, about a third of patients with epilepsy have seizures refractory to pharmacotherapy [2,3]. Patients with DRE have an increased risk of premature death, injury, psychosocial dysfunction, and decreased quality of life, so the development of more effective treatment methods is an urgent clinical need [1]. However, different types of epilepsy and seizures, as well as complex patterns of resistance to AEDs complicate the problem [2]. Currently, drug resistance is a key problem in the theory and practice of epileptology [1]. The International League Against Epilepsy (ILAE) defines this concept as follows: “…drug-resistant epilepsy is defined as failure of adequate trials of two tolerated, appropriately chosen and used antiepileptic drug schedules (whether as monotherapies or in combination) to achieve sustained seizure freedom…” [2].

AEDs act on different molecular targets and selectively alter the excitability of neurons. Thus, the neuronal activity associated with seizures is blocked without disrupting the normal activity necessary for the transmission of signals between neurons. The membrane potentials depend on the ratio of the activity of the anionic and cationic channels of the membranes, each of which opens at a certain time. The speed of channel functioning is affected by ligand binding (neurotransmitters) and changes in membrane voltage [3]. Both excitatory and inhibitory neurotransmitters are involved in synaptic signal transmission. The main inhibitory neurotransmitter is gamma-aminobutyric acid (GABA), and the excitatory one is glutamate (Glu). Drugs that have an antiepileptic effect act on one or more target molecules in the brain. The targets are not only ion channels or neurotransmitters but also enzymes that metabolize neurotransmitters (GAD). Antiepileptic activity is achieved by changing the activity of neurons and disrupting the synchronization of neuronal circuits [4,5].

The action of AEDs, for example, benzodiazepines and barbiturates, can be mediated through a change in secretion or binding to the receptor of the main inhibitory neurotransmitter GABA. Felbamate and topiramate also partially act through GABAA receptors. The possible targets of AEDs acting by this mechanism are ionotropic receptors Glu, N-methyl-D-aspartate (NMDA), and kainate located on GABA-ergic neurons.

At the moment, most AEDs affect GABA receptors (benzodiazepines and phenobarbital), Glu metabolism (valproates, vigabatrin, and lamotrigine), and Na channels and Ca channels (lamotrigine, topiromate, and ethosuximide) [4].

Drug resistance may occur or disappear during epilepsy or its treatment, and among most patients, drug resistance appears to be permanent and occurs de novo [6]. Still, the question remains unclear—why and how does epilepsy become resistant to medications, while other patients with seemingly identical types of seizures can control the seizures with the help of anticonvulsants? In recent years, several suspected mechanisms underlying drug resistance in epilepsy have been identified. Based on experimental and clinical studies, two main neurobiological theories have been put forward: (a) the removal of AEDs from epileptogenic tissue due to the overexpression of the carriers of many drugs and (b) the reduction in sensitivity to the target drug in epileptogenic brain tissue [6]. From a clinical point of view, genetic and clinical features as well as structural brain lesions are associated with DRE [6,7,8]. To achieve a therapeutic effect, the availability and preservation of structures affected by drugs are very important.

There are many factors that can contribute to the development of DRE, including genetic, epigenetic, neurological, immunological, and environmental factors that can affect the concentration of the drug in the brain or the response to AEDs [7,8].

It is generally recognized that the effectiveness of AEDs is determined by their ability to overcome the BBB and bind to intraparenchymal targets [9,10,11]. According to one hypothesis, drug resistance occurs when target sites are structurally and/or functionally modified in such a way that they become less sensitive to AEDs [3,4]. A large number of targets for AEDs have been identified in the brain, many of which undergo molecular changes in epilepsy [7,8,9]. It is possible that damage to the BBB may affect resistance in DRE.

The barrier function is the result of a combination of a physical barrier (tight junctions between cells that reduce the flow through intercellular contacts or the paracellular pathway), a transport barrier (specific transport mechanisms mediating the dissolved flow), and a metabolic barrier (enzymes that metabolize molecules during transit). It is not fixed but can be modulated and regulated both in physiology and pathology [10].

In the brain and spinal cord of mammals, including humans, the BBB is created primarily by endothelial cells that form the walls of capillaries [11]. However, for the development and maintenance of the BBB, as well as the modulation of its function, pericytes, astrocytes, and the basement membrane are important (proteins of the extracellular matrix of the basement membrane, secreted by endothelial cells (endothelial basement membrane) and pericytes/astrocytes (parenchymal basement membrane)). In this regard, the study of structural changes is important for assessing the function of BBB cells. Although it is now generally recognized that VEGF is enhanced in epilepsy, there are many questions about the mechanisms of its regulation, the relationship with other factors that trigger this increased reaction, and so on [12].

Neuroinflammation in epilepsy foci, which is combined with the development of glioneuronal apoptosis, affects angiogenesis and BBB permeability and creates conditions for the development of drug resistance and the progression of epilepsy [13,14].

Patients with epilepsy have impaired BBB function, which may reduce the control of epileptic seizures when taking AEDs [12,15]. Studies show that changes in the BBB can lead to a decrease in the concentration of AEDs in the brain and an increase in their toxicity in other organs, which can reduce their effectiveness and lead to drug resistance [16].

It is assumed that BBB damage contributes to the development of drug-resistant seizures in three ways. The direct effect of inflammatory mediators on the vascular endothelium of the brain, including the direct destruction of dense contacts between endothelial cells, induces oxidative stress and abnormal angiogenesis (the formation of “leaky” vessels) [12]. Through this type of BBB damage, serum albumin penetrates into the nervous tissue, which normally should not be present there. Albumin enhances the effect of drug binding, thereby reducing the functionally significant levels of unrelated drugs in the target areas of the brain [15,16]. Another mechanism that stimulates the synthesis of inflammatory mediators in damaged endothelial cells is P-glycoprotein. The increased outflow of AEDs leads to a decrease in the absorption of the drug by the brain and, as a result, to conditional resistance [10,14]. The third way involves the modification of voltage-dependent ion channels by inflammatory mediators penetrating through the damaged BBB, which leads to a decrease in the sensitivity of these receptors to the drugs [13].

Thus, damage to the BBB may be one of the possible causes of resistance in DRE, and further research in this area may help develop new treatments for such patients [1,17].

In this regard, the aim of this work was to assess the damage and dysfunction in the BBB in the area of epileptic foci in patients with DRE under conditions of neuroinflammation.

## 2. Results

This section may be divided into subheadings. It should provide a concise and precise description of the experimental results, their interpretation, as well as the experimental conclusions that can be drawn.

### 2.1. VEGF Expression Content in Brain Capillary Endotheliocytes

**Figure 1:**
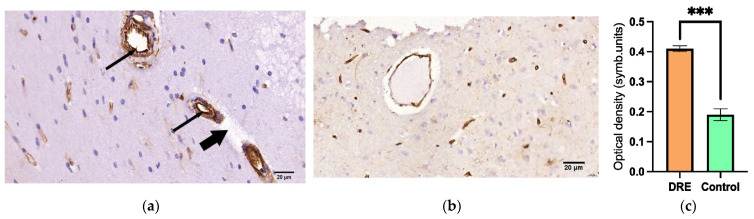
Morphological changes in the white matter of the brain: (**a**) moderately expanded perivascular space (thick arrow), a pronounced VEGF-positive reaction (IHC staining), and (thin arrows) a patient with DRE; (**b**) no VEGF-positive reaction in the Control 1 group of patients without epilepsy; (**c**) optical density of VEGF-positive cells in the DRE group (orange bar) vs. Control 1 group (green bar) (***—*p* < 0.001). IHC—immunohistochemistry; DRE—drug-resistant epilepsy; VEGF—vascular endothelial growth factor. VEGF-A antibody staining, hematoxylin staining, magnification ×400.

All patients in the DRE group showed a pronounced expression of VEGF-A, localized mainly in the cytoplasm of endotheliocytes, moderately pronounced perivascular edema, and thickening of the capillary wall (Figure 1). The optical density (symb.units) of VEGF-positive cells in the DRE group was 0.41 ± 0.01 versus 0.19 ± 0.02 in the Control 1 group (*p* < 0.001).

### 2.2. Blood Serum Cytokines

In the studied blood serum samples, the contents of pro-inflammatory interleukin (IL)-1β and IL-1RA did not differ significantly from their content in the Control group 2 (Table 1). In the DRE group, the concentrations of IL-2 (*p* < 0.001), IL-8 (*p* < 0.001), and EGF (*p* < 0.05) were significantly lower than in the Control 2 group. At the same time, the levels of tumor necrosis factor (TNF)-α (*p* < 0.05), IL-4 (*p* < 0.01), IL-5 (*p* < 0.05), IL-7 (*p* < 0.001), GRO (*p* < 0.001), and VEGF (*p* < 0.01) were significantly higher in the DRE group than in the control group. The levels of anti-inflammatory cytokine IL-10 and interferon (IFN)-γ did not differ significantly between the groups.

### 2.3. The Content of Autoantibodies in Blood Serum

**Figure 2:**
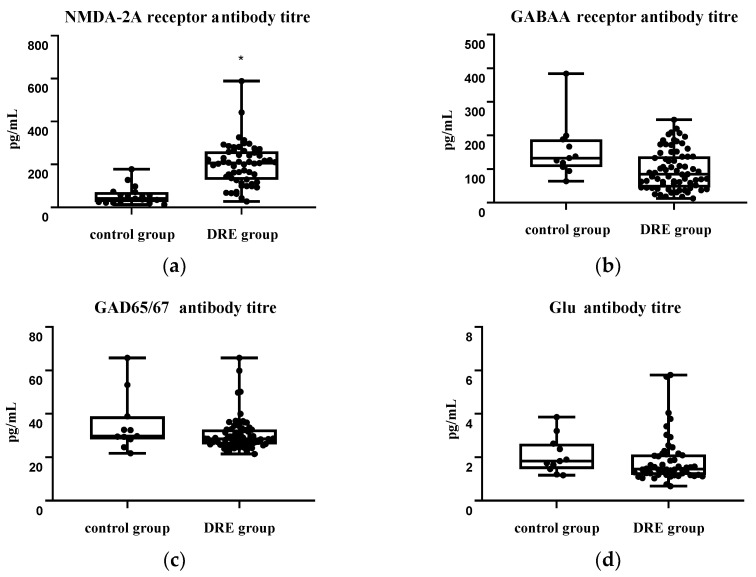
The level of antibodies in the blood serum of patients with DRE compared with the control group: (**a**) NMDA-2A receptor; (**b**) GABA A receptor; (**c**) GAD65/67; (**d**) Glu. DRE group vs. Control 1 group (*—*p* < 0.001).

According to the results of solid-phase ELISA, our patients showed a statistically insignificant decrease in the titer of antibodies to glutamate (Glu) receptors; however, at the same time, a significant increase in the titer of antibodies to NMDA-2A receptor was detected compared with the control group. No differences were found in the titer of autoantibodies to glutamate decarboxylase (GAD) and to GABA receptors (GABA-R) (Figure 2).

### 2.4. Ultrastructural (TEM) Examination of the Vascular Wall of the Capillaries

**Figure 3:**
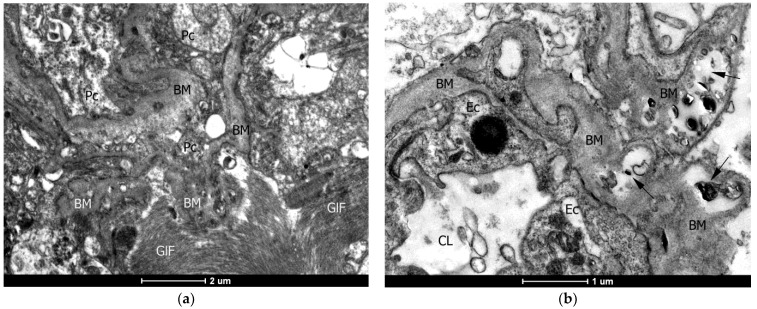

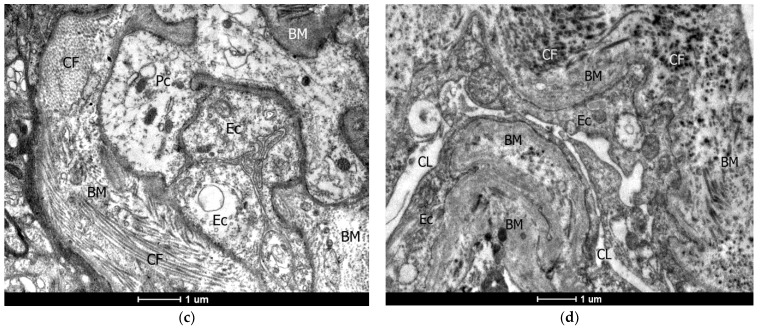
Fragments of capillaries with unevenly thickened areas of the basement membrane (BM). (**a**) Bumpy, in some places sharply expanded BM limits pericytes (Pc) with hyperchromic cytoplasm. From the outside, powerful bundles of gliofilaments (GlF) are adjacent to the capillary. (**b**) Loose, swollen BM has foci of encrustation with destroyed membranes and calcifications (arrows). (**c**) Cross-section of the capillary: pronounced expansion of the BM also occurs due to the germination of collagen fibers (CF) in it, causing asymmetry of the vessel; fibrosis is observed in some Pc. (**d**) A longitudinal section of the capillary in which the transverse growth of the BM and its powerful fibrosis led to a critical narrowing of the lumen. CL—capillary lumen; Ec —endotheliocyte. Electronograms: (**a**)—×8200; (**b**)—×11,500; (**c**,**d**)—×9900.

Among the destructive and pathological changes in the vascular wall of capillaries, an unevenly pronounced thickening of individual areas of the basement membrane was the most common (Figure 3).

Pronounced thickening of the basement membrane in combination with the germination of collagen fibers in it, accumulation of calcifications, and fibrosis of pericytes can disrupt the permeability and plasticity of the vascular wall, which can cause microcirculation disorders and cause hypoxia in this region.

**Figure 4:**
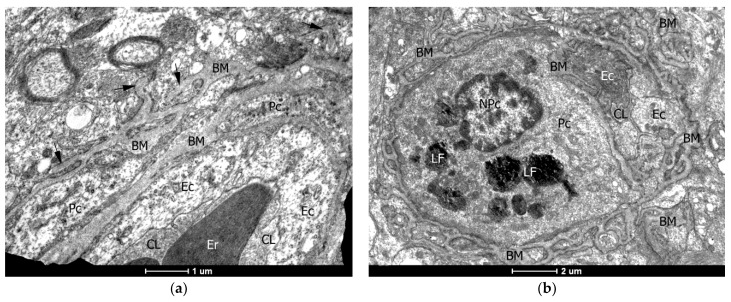

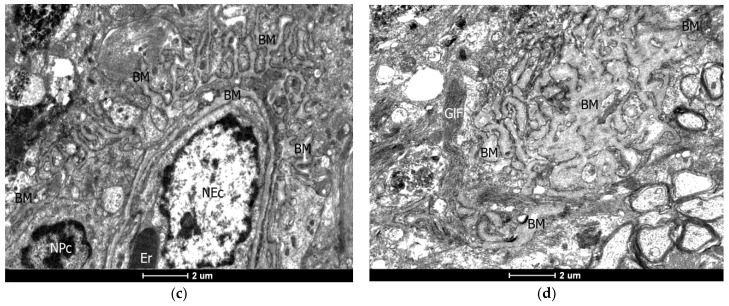
Malformed growth of the basement membrane of the capillaries of the brain. (**a**) A small capillary with erythrocytes (Er) in the capillary lumen (CL) is surrounded by a basement membrane (BM), uneven in the thickness and density of its matrix. The arrows indicate the BM processes that are growing into the surrounding brain tissue. Endotheliocytes (Ec) and pericytes (Pc) are dystrophic, with a minimal set of cytoplasmic organelles. (**b**) A small capillary with a narrowed CL and a large Pc containing lipofuscin granules (LF) is surrounded by a network of branched BM. (**c**) A fragment of capillaries with Er in the lumen and a large edematous nucleus of the endotheliocyte (NEc). The BM is multilayered, and its outer zone creates a picture of a radiant crown with numerous parallel, sometimes branched processes. They are not always close together, creating pericytic cavities, which indicates a dynamically developing process of formation of vascular malformations. (**d**) A malformation-like tangle of multiple, branched, sometimes merged into a single shapeless conglomerate, processes of the BM. GlF—gliofilaments; NPc—nucleus of the pericyte. Electronograms: (**a**)—×11,500; (**b**)—×16,500; (**c**,**d**)—×6000.

The combination of an unevenly expanded basement membrane with the tendency of its germination into the parenchyma of the brain was not infrequently noted, which apparently led to an increase in loops with compartments of pericytes and, thus, a thickening of the vascular wall (Figure 4).

## 3. Discussion

### 3.1. Damage to the Permeability of the BBB

#### 3.1.1. VEGF

VEGF in the systemic circulation plays the role of a mitogen for the epithelial cells of blood and lymph vessels. It is known that its mRNA transcription is induced by such cytokines as PDGF (platelet-derived growth factor), EGF (epidermal growth factor), TNF-α, TGF-β1 (transforming growth factor beta 1), and IL-1β [18]. The basic contents of the VEGF-A receptor in organs and VEGF in the blood are rather low. The circulation of a large amount of VEGF has a strong effect on vascular permeability and has a powerful angiogenic effect, which is important for neovascularization processes in pathological situations [19]. Among patients from the DRE group, a high concentration of VEGF in the blood serum was detected. Elevated levels of freely circulating VEGF protein in the blood induce endothelial hyperpermeability by direct action on endothelial cells [20].

#### 3.1.2. EGF

Among DRE patients we observed a lower content of EGF, which is responsible for the growth and division of endothelial cells, in combination with a high level of VEGF, which may indicate an imbalance between the function of angiogenesis and the permeability of cerebral vessels, which are structural units of the BBB. Elevated levels of the VEGF-A receptor found in cerebral microvessels among DRE patients may affect major structural components of tight junctions [21]. The formation of tight junctions and regulation of BBB permeability is carried out by astrocytes secreting chemokines such as EGF, TGF-β, GRO, and VEGF, which act on endothelial cells [22,23]. At the same time, high concentrations of TGF-β, GRO, and VEGF are recorded in the systemic circulation, and the entry of these cytokines into the plasma may be due to impaired function of cerebral vascular endotheliocytes.

#### 3.1.3. VEGF-A and Chemokines

Increased synthesis of chemokines by astrocytes against the background of an increase in VEGF-A expression by endotheliocytes may indicate damage to the structural integrity of the functional units of the BBB and, as a result, increased BBB permeability. Some authors have described BBB permeability as one of the determinants of a medicine’s bioavailability and resistance to various AEDs which is typical for patients with DRE [24].

#### 3.1.4. Cytokines in the Blood and Neuroinflammation

Structural damage to the BBB may be the cause of the appearance of pro-inflammatory cytokines in the blood, in the absence of other possible causes of cytokine increase in DRE patients. The circulation in the bloodstream of such cytokines as TNF-α, IL-1, and IL-8 usually indicates the acute phase of the body’s response to inflammation. Neuroinflammation is regulated by both pro-inflammatory cytokines (IFN-γ) and inflammation inhibitors (IL-10) [25]. Our results demonstrate normal levels of anti-inflammatory IL-10, with elevated TNF-α and reduced IL-2 compared to the control group.

In the studied blood plasma samples, the content of pro-inflammatory IL-1β and its natural anticonvulsant antagonist IL-1RA did not differ significantly from their content in the group of patients without epilepsy. However, there are data indicating a decrease in IL-1RA/IL-1β after an attack [26]. Such differences may be due to the fact that we took blood during the seizure-free period established in the hospital, while other researchers take blood for analysis immediately after a seizure or after a certain time.

IL-2 is an important immunoregulatory cytokine that is expressed by cells of the immune system and the brain [27]. It is known that IL-2 promotes the regeneration of neurons after their damage, and also stimulates the proliferation and differentiation of oligodendrogliocytes. In our study, the level of IL-2 in the blood of patients with DRE was reduced. IL-2 deficiency in the blood of DRE patients may be one of the factors reducing a drug’s bioavailability, which depends on the function of the BBB [28].

We found an increase in the IL-4 level, which is involved in balancing neuroinflammation, which may indicate a response to the appearance of TNF-α in the blood and a slowdown in the synthesis of primary response cytokines. Reduced levels of the angiogenesis stimulator IL-8 in serum may indirectly indicate its functional deficiency [29], which developed as a result of the chronicization of the epileptic process. The decrease in IL-8 may promote increased adhesion of neutrophils to endothelial cells already activated by proinflammatory cytokines released by brain cells.

Against the background of IL-8 decrease, increased expression of VEGF in the systemic circulation promotes the mobilization of inflammatory cells to the locus of damage, supporting the local inflammatory process [30]. From our previous results, it can be seen that in the cortex of the temporal lobe of DRE patients, neuroinflammatory processes occur, including a high expression of TNF- α and FAS by immunocompetent cells of the nervous tissue. In the white matter, these processes are also present but less intense [13]. At the same time, there is the previously shown increased lymphocytic infiltration. T-lymphocytes make up the bulk of the infiltrates, including T-killers, B-lymphocytes, and macrophages, which indicates the active participation of cellular immunity reactions in the pathogenesis of epilepsy [31]. Thus, increased production of pro-inflammatory cytokines in the nervous tissue contributes to a local increase in BBB permeability.

The increase in the content of pro-inflammatory TNF-α and IL-7 in the systemic circulation of DRE patients, as well as the compensatory increase in the concentration of anti-inflammatory IL-4, which we found, probably reflects the response of innate immunity and endothelial cells associated with brain tissue damage [32]. Further research in this area will help identify a new therapeutic target in the treatment of DRE.

#### 3.1.5. Autoantibodies to GAD, GABA, and NR2 NMDA Receptors

Based on the results of solid-phase ELISA, we revealed an increase in the titer of autoantibodies to NR2 NMDA in DRE patients compared with the control group. The absence of an increase in antibodies to GABAA, Glu, and GAD indicates the absence of an immune response to the “exposure” of these targets when the BBB is damaged. However, the increase in antibodies to NMDA-2A receptors indicates that the immune system has access to targets in the nervous tissue, which also confirms damage to the BBB. At the same time, autoaggression to NMDA-2A may indirectly affect the development of resistance to AEDs. The known mechanism of action of some drugs (felbamate) is associated with blocking NMDA receptors [3]. The mechanism of action of some other drugs is not fully known, and NMDA receptors may also be involved in it.

Based on our findings, we have observed that the following factors indicate damage to BBB permeability:

- Increased levels of VEGF, GRO, and EGF deficiency in plasma, combined with overexpression of the VEGF-A receptor by endotheliocytes;
- Penetration of pro-inflammatory factors and autoantibodies to NR2 NMDA receptors in the blood in the absence of systemic inflammation in patients with DRE;
- The absence of changes in the titer of autoantibodies to GAD and to GABA receptors in the blood of patients with DRE indicates the preservation of the targets of AEDs acting on these receptors.

### 3.2. Basal Membrane and Genetic Alterations

A change in the basement membrane is observed in hypoxia, traumatic brain lesions, astrocytic tumors with active endothelial proliferation, and aging [33,34,35].

The process of hypoperfusion in the zone of the epileptic foci triggers the mechanisms of proliferation of endothelial cells and barrier formation, which can lead to the development of an imbalance between these structures. It has been shown that both patients with temporal lobe epilepsy and animal models of epilepsy exhibit the formation of protrusions or strings of the basement membrane of blood vessels [14]. It is a well-known biological phenomenon of stromal vascular hyperplasia in chronic inflammation and damage with the formation of hypertrophic scars and inflammatory polyps, accompanied by pronounced angiogenesis and hyperproduction of collagen. Excessive collagen production is associated with the overexpression of VEGF, TNF-α, and an overall increase in scar volume [36,37].

In the foci of epileptogenesis against the background of chronic neuroinflammation, a similar situation develops with the phenomena of hyperplasia of the basement membrane [12,13,38]. The abnormal growth changes in the basement membrane have a dysplastic character, which suggests a genetic basis for these changes. It is possible that such vascular changes may be the beginning of the formation of foci of angiomatosis and cavernomas, which are detected in epilepsy [12,14,39].

The basement membrane is the complex of space-organized proteins (collagen IV, III, VII, laminin, etc.) with 2D and 3D structures [40]. The production of this complex is under the control of many factors including VEGF.

The key element of the basement membrane is collagen IV. It associates with laminin, entactin, and heparan sulfate proteoglycans to form sheet-like basement membranes that separate the epithelium from connective tissue. This protein exhibited not fibrillar, but 2D net structures. The collagen IV molecule is a heterotrimer of two alpha-1 chains and one alpha-2 chain [41]. However, six types of collagen IV alpha chains are described. The *COL4A1* and *COL4A2* genes are situated at *13q34* and have a common, bidirectional promoter [42]. These genes are associated with different forms of brain small vessel disease and susceptibility to intracerebral hemorrhage [43,44]. Laminin is a basement membrane protein composed of three nonidentical chains arranged in a cross-shaped structure, but mutations in this group of genes are not associated with cerebral pathology according to available data.

Previously described malformations of the basement membrane may be the result not of mutation but incorrect folding. Insufficient folding of type IV collagen and formation of abnormal basement-membrane-like structure is described, but not in brain vessels [45]. Laminin-111 (three-chain structure, encoded by *LAMA1*, *LAMB1*, and *LAMB2*) was found to be important for correct epithelial basal folding in a zebrafish experimental model [46].

The possibility of the association of collagen genes with epilepsy has also been described [47].

Thus, the described malformation of the basement membrane may be caused by:

1. Mutations in *COL4A1* and *COL4A2*, regular or mosaic (more possible);
2. Incorrect folding as a possible result of the incorrect functioning of the laminin-111 pathway with secondary inflammation;
3. Disorder in external regulation (VEGF, *inflammation*) with secondary local disorders in collagen post-translation modifications and folding.

## 4. Materials and Methods

### 4.1. Study Design and Patients

This study followed a case–control design. Biopsies of 30 patients with focal DRE (17 men and 13 women; mean age of patients: 27.1 ± 4.7 years; 14 with left-sided lesions, 16 with right-sided lesions) were studied. All patients were treated at Polenov Neurosurgical Institute, Almazov National Medical Research Centre (ANMRC), St. Petersburg, Russia (2017–2022). The work was carried out according to the principles of voluntariness and confidentiality in accordance with the Federal Law “On the Basics of Health Protection of Citizens in the Russian Federation” 21 November 2011 N 323-FZ and the Helsinki Declaration on Human Rights, and approved by the ethical committee of ANMRC. Written consent was obtained from the subjects. The pre-surgical stage of diagnostics was carried out according to the algorithm of the standard diagnostic complex for examining DRE patients, including clinical observation, the study of neurological, neuropsychological, and mental status, and electrophysiological and neuroimaging investigations. The type of epileptic seizures was established in accordance with the International League Against Epilepsy (2017). The patients with complex partial seizures with secondary generalization prevailed, while simple partial seizures were much less present.

The patients were found to have atrophy mainly of the frontal and temporal lobes, gliosis and cystic-gliosis changes in the brain, focal cortical dysplasia, and hippocampal sclerosis. The patients underwent anterotemporal resections, and resections of cortical epileptic focus under the electrocorticography control. Biopsies of the cortex and white matter of the temporal lobe obtained intraoperatively served as material for electron microscopic, histological, and immunohistochemistry (IHC) analyses.

The material of the comparison group for histological study, IHC (cortex and white matter of the temporal lobe) was obtained during autopsies (Control 1), in the first 6 h after death from 10 patients who died from somatic diseases, such as acute myocardial infarction, gastric ulcer complicated by bleeding, mesenteric thrombosis, and pulmonary embolism. These patients had no history of neurological disorders.

To detect the levels of cytokines and chemokines, blood plasma samples were taken from 20 DRE patients (20–32 years old) without acute or chronic inflammatory diseases. The comparison group included 12 healthy volunteers without acute or chronic inflammatory diseases, with a mean age of 28 ± 4.4 (Control 2), who signed an informed voluntary consent form, and they also did not eat for 8 h before the study.

All studies of biopsy specimens and blood serum were carried out at the Laboratory of Comparative Biochemistry of Cell Functions and Collective Equipment Center of the Institute of Evolutionary Physiology and Biochemistry, Russian Academy of Sciences.

The data that support the findings of this study are available on request from the corresponding author. The data are not publicly available due to privacy or ethical restrictions.

### 4.2. Transmission Electron Microscopy (TEM)

Temporal lobe biopsies from 10 patients with DRE were fixed in a mixture of 4% paraformaldehyde and 0.5% glutaraldehyde in 0.1 M cacodylate buffer (pH 7.2–7.4) cooled to 4 C. Additional fixation was carried out with 1% osmium tetroxide, and then the samples were dehydrated and embedded in a mixture of epoxy resins (araldites). Ultrathin 50–60 nm sections were prepared on an Ultratome LKB-III (LKB-Producter AB, Stockholm-Bromma 1, Sweden). The morphology was observed and recorded using a TEM FEI Tecnai G2Spirit BioTWIN (FEI Europe B.V., P.O., Amsterdam, The Netherlands), at an accelerating voltage of 80 kV, provided by the Centre of Collective Use of Sechenov Institute of Evolutionary Physiology and Biochemistry, Russian Academy of Sciences.

### 4.3. IHC

IHC studies were performed on 20 patients with DRE for the analysis of VEGF-A content in brain capillary endotheliocytes. Biopsies of the temporal lobe were fixed with 10% paraformaldehyde in 0.1 M sodium phosphate buffer, dehydrated in a standard manner, and embedded in paraffin. IHC reactions were performed on paraffin-embedded 5–7 μm thick slices of the brain temporal lobe biopsies according to the standard protocol. The VEGF-A antibody (Anti-VEGF Receptor 2 antibody [SP123] Abcam, Boston, MA, USA) was used as a primary antibody. For visualization, the Streptavidin-Peroxidase Polymer Ultrasensitive system (Streptavidin-Peroxidase Polymer, Ultrasensitive, Product Number: S 2438, Sigma-Aldrich, St. Louis, MO, USA) and DAB chromogen (3,3′-Diaminobenzidine tetrahydrochloride hydrate, Product Number: D5637, Sigma-Aldrich, USA) were used. The sections were counterstained with Gill’s hematoxylin, and embedded in Bio Mount HM synthetic embedding medium (Bio Mount HM Mounting medium, Catalog number: 05-BMHM100, BIO-OPTICA Milano, Italy). Additionally, reactions lacking primary antibodies were performed to ensure the specificity of the observed staining. Sections were analyzed with a light microscope (Zeiss Mirax Midi BF/FL Fluorescence Slide Scanner Imaging System, Carl Zeiss MicroImaging GmbH., Jena, Germany).

### 4.4. Multiplex Analysis

Research using Luminex MagPix (MAGPIX^®^, EMD Millipore Corporation, Billerica, MA, USA) has been conducted according to the manufacturer’s recommended standards and protocol on the equipment provided by CEC IEPB, RAS. All samples and standards were placed in two wells for each sample. Detection was carried out using a streptavidin–phycoerythrin solution. MILLIPLEX^®^ map human cytokine/chemokine 35-Plex Panel (MILLIPLEX MAP Human Cytokine/Chemokine Magnetic Bead Panel-Immunology Multiplex Assay, Catalog number: HCYTOMAG-60K, Thermo Fisher Scientific Inc., Vienna, Austria) was used: EGF, Eotaxin, FGF-basic, G-CSF, GM-CSF, GRO, IFN-alpha, IFN- γ, IL-1 beta, IL-1 alpha, IL-1RA, IL-2, IL-2R, IL-3, IL-4, IL-5, IL-6, IL-7IL-9, IL-12 (p40/p70) IL-13, IL-15, IL-17A, IL-17F, IL-22, IP-10, MCP-1, MIG, MIP-1 alpha, MIP-1 beta, RANTES, and VEGF.

A total of 20 patients, 2–3 days before surgery, did not eat for 8 h before the study, against the background of an absence of seizures, and blood serum samples were taken. Blood samples were not taken from all patients, as 10 patients had frequent seizures and no seizure-free period was recorded.

Part of the serum was used for the quantitative determination of autoantibodies. We analyzed the content of antibodies to GAD (AED245Hu ELISA Kit for Anti-Glutamic Acid1 set 78,969.00 Decarboxylase Antibody (Anti-GAD)), antibodies to GABA receptors (Human anti-gamma-aminobutyric acid A receptor antibody (anti-GABAA receptor)), antibodies to NMDA receptors (AEE806Hu 96 Tests Enzyme-linked Immunosorbent Assay Kit For Anti-Glutamate Receptor, Ionotropic, N-Methyl-D-Aspartate 2A (Anti-GRIN2A)), and antibodies to glutamate (AES122Ge 96 Tests Enzyme-linked Immunosorbent Assay Kit For Anti-Glutamic Acid (Anti-Glu)). The study was conducted on an Immulite 1000 immunochemiluminescent analyzer (Immulite 1000, Siemens Healthcare GmbH, Erlangen, Germany) using kits from the manufacturer CLOUD-CLONE CORP (CLOUD-CLONE CORP., Wuhan, Hubei, China). Biological material was taken in the morning.

### 4.5. Statistical Analysis

Statistical analysis was carried out using Student’s *t*-test (*p* < 0.05).

IHC. The positive IHC staining of endotheliocytes in the temporal lobe sections was evaluated by calculating the optic density of stained cells relative to the background areas in 10 fields of view using the PhotoM 1.21 program.

Multiplex analysis. The normality test for all studied parameters was performed using the Shapiro–Wilk criterion. Quantitative indicators corresponded to the normal distribution and were presented in the form of an average value and a standard deviation, and those not corresponding were presented as the median and interquartile range (25–75 percentiles). A comparison of quantitative data was carried out using Student’s unpaired criterion. Statistical data processing was carried out using GraphPad Prism 8.0.1.

## 5. Conclusions

In the epileptic focus in the tissue of the temporal lobe of patients with DRE, the following mutually potentiating pathological processes occur, leading to the destruction of the BBB and damage to its permeability in conditions of chronic neuroinflammation:

1. Damage to BBB permeability in conditions of neuroinflammation is caused by endotheliocyte dysfunction with activation of angiogenesis (VEGF overexpression), stimulating the hyperproduction of basement membrane’s collagen and insufficiency of endothelial proliferation factors (EGF reduction);
2. Observed malformation-like transformation of the basement membrane of the microcirculatory bed complicates the delivery of AEDs. Dysplasia of the basement membrane is the result of inadequate reparative processes in chronic inflammation.

In addition, the absence of changes in the titer of autoantibodies to GAD and GABA receptors in the blood of patients with DRE reduces the likelihood of developing resistance to drugs acting on these receptors. The detection of autoantibodies to NR2 NMDA receptors in the blood confirms damage to the BBB and may also contribute to resistance.

In conclusion, it should be noted that damage to the microcirculatory network of the brain should be considered one of the leading factors contributing to DRE.

Further study of this issue should open up new perspectives on understanding the mechanisms of the development of DRE and the development of alternative treatment strategies.

## Figures and Tables

**Table 1:** The level of cytokines and chemokines in the serum of patients with DRE compared to the Control 2 group.

Cytokines, pg/mL	DRE Group	Control 2	*p*-Value
IL-1β	1.26 ± 0.33	1.32 ± 0.41	ns
IL-1RA	32.99 ± 4.147	32.36 ± 6.49	ns
IL-2	0.978 ± 0.28	4.81 ± 0.71	*p* < 0.001
IL-4	43.73 ± 2.57	13.35 ± 3.30	*p* < 0.01
IL-5	43.73 ± 2.57	22.37 ± 5.80	*p* < 0.05
IL-7	16.65 ± 3.07	8.13 ± 1.67	*p* < 0.001
IL-8	14.04 ± 1.46	26.13 ± 3.80	*p* < 0.001
IL-10	5.61 ± 0.89	4.31 ± 1.15	ns
TNF-α	33.09 ± 1.23	24.85 ± 1.32	*p* < 0.05
VEGF	316.1 ± 55.28	95.22 ± 15.78	*p* < 0.01
GRO	3054 ± 200.8	1367 ± 187.3	*p* < 0.001
EGF	43.72 ± 5.63	83.62 ± 24.06	*p* < 0.05
IFN-γ	13.45 ± 2.05	14.17 ± 2.20	ns

Note. ns: not significant.

## Data Availability

The data presented in this study are available on request from the corresponding author. The data are not publicly available due to privacy concerns.
